# Prevalence of non-typhoidal *Salmonella* and risk factors on poultry farms in Chitwan, Nepal

**DOI:** 10.14202/vetworld.2021.426-436

**Published:** 2021-02-20

**Authors:** Sumit Sharma, Peter D. Fowler, Dhan Kumar Pant, Subir Singh, Melinda J. Wilkins

**Affiliations:** 1 Agriculture and Forestry University, Faculty of Animal Science, Veterinary Science and Fisheries, Department of Veterinary Physiology and Biochemistry, Rampur, Chitwan 44200, Nepal; 2 Michigan State University, College of Veterinary Medicine, Department of Large Animal Clinical Sciences, 736 Wilson Rd. East Lansing, Michigan, 48824, USA; 3 National Zoonoses and Food Hygiene Research Centre G.P.O. Box: 1885 Jeevan Smriti Marg, Chagal, House No. 468/32, Ward No. 32, Kathmandu, 44600, Nepal; 4 Agriculture and Forestry University, Faculty of Animal Science, Veterinary Science and Fisheries, Department of Veterinary Medicine and Public Health, Rampur, Chitwan, 44200, Nepal

**Keywords:** farm risk factors, Nepal, non-typhoidal *Salmonella*, poultry, *Salmonella enterica*

## Abstract

**Background and Aim:**

Poultry is becoming an increasingly important source of protein in the Nepalese diet. The Chitwan region of Nepal is the hub of the emerging poultry industry. Little is known about the prevalence of non-typhoidal *Salmonella* (NTS) on poultry farms or the role of farm management practices that may contribute to the presence of NTS on farms. The role of poultry in the transmission of *Salmonella enterica* to humans is also poorly defined. This descriptive study seeks establish baseline data through estimation of the prevalence of NTS on broiler and layer operations in various farms of the Chitwan district of Nepal.

**Materials and Methods:**

Based on district documents on poultry production and meat marketing, a purposive sampling of 18 commercial poultry farms comprising ten broilers farms and eight layers farms was conducted. Environmental samples including water, litter, feces, feed, farm, and eggshell swabs were randomly collected from each farm. Samples were cultured and tested for the presence of NTS; positives were serotyped, and antimicrobial susceptibility determined. A comprehensive farm and practice questionnaire was administered to each farm manager.

**Results:**

The farm level point prevalence rate was 55% (10 of 18 farms) for *S. enterica*. Of the total 288 farm environmental samples collected, 26 samples (9%) were positive. The rate of isolation varied according to the origin of samples: Water (27.5%), feces (10.6%), litter (8.6%), farm swabs (5%), feed (1.8%), and eggshells (0%). Farm management variables/risk factors are summarized and categorized as non-modifiable and modifiable for analysis. Broiler operations were more likely to be positive than layer operations as were poultry houses with two or less open sides. All-in/all-out management style was found to be protective. Due to the small sample size (18 farms), no associations reached statistical significance.

**Conclusion:**

Based on environmental sampling results, NTS is highly prevalent on the poultry farms in the Chitwan district of Nepal. Certain risk factors are associated with finding NTS on farms. Our findings are generally in agreement with other studies in similar countries with rapidly emerging poultry industries. The identification of risk factors provides owners, technicians, and veterinarians with some guidance to help reduce the prevalence of NTS on farms. This baseline data are critical to understanding the epidemiology of zoonotic strain of NTS in the region and are necessary for the design of future studies and mitigation plans and underlines the need for a one-health approach to protect public health-related to *Salmonella* spp. from poultry farms.

## Introduction

Non-typhoidal *Salmonella* (NTS) infections are estimated to cause around 153 million cases of gastroenteritis and 57,000 deaths globally each year, making it one of the leading causes of bacterial diarrhea worldwide [1]. Infections of humans with NTS are frequently associated with the consumption of contaminated food and are considered the second largest cause of food-borne illnesses after *Campylobacter* species [2-5]. This highlights the need for a global effort with a management and monitoring framework to control antibiotic resistance to protect public health related to *Salmonella* spp. from poultry farms. Although several *Salmonella enterica* serovars are consistently found at a high incidence within the poultry industry, geographic and temporal variation play a prominent role in the distribution [6,7]. Poultry is increasingly playing a major role in the human food chain. Due to increased globalization, modern poultry industries have created more complex opportunities for the spread of *S. enterica*, especially linked to international travel, livestock trade, and human migration [6-10].

Although there has been extensive research on *S. enterica* spp. among poultry farms globally, there is a lack of data in developing countries particularly in South Asia, including Nepal. Nepal is an agriculture-based country with around two-thirds of the population depending on agriculture for their livelihood. The livestock sector has contributed 26.8% of agricultural gross domestic production (AGDP) and 11% of gross domestic production (GDP). Poultry alone contributed 3.5% of GDP in 2014 [11,12] and 4.0% in 2020 with worth over NRs 50 billion [13]. Annual per capita meat consumption from all livestock and poultry in Nepal increased from 10.2 kg (in 2002) to 12.2 kg in 2011 [11] driving the rapid expansion of the poultry industry. According to the Food and Agriculture Organization data based on imputation methodology, the production of poultry meat increased rapidly from 20 million chickens to over 77 million from 2008 to 2018, almost a four-fold increase [14]. Due to the economic importance of this sector and the increasing reliance on poultry as a source of nutrition, food-borne pathogens such as *S. enterica* are of increasing concern as they pose a potential threat to both livestock production and human health. Despite this expansion, very little research has been conducted at the farm level regarding the risk factors which may increase the chances of acquisition, spread, and maintenance of NTS on the poultry farms in Nepal.

This study aims to establish baseline data through estimation of the prevalence of NTS on broiler and layer operations in various farms of the Chitwan district of Nepal. Farm-level management practices that may contribute to acquisition, spread, and maintenance of NTS were collected as well as environmental samples from 18 farms in Chitwan, Nepal’s leading poultry producing district.

## Materials and Methods

### Ethical approval and informed consent

Although the village and district of each farm were recorded, the identity and exact location of each farm were coded to ensure anonymity. Oral permission was obtained from the owner of each poultry farm before collecting environmental samples from the farm. No live animals were touched or harmed in this study. This study was reviewed by the Michigan State University Institutional Animal Use and Care Committee and the Human Subject Review Committee and ruled “Exempt” from review by both Boards.

### Study area and period

**Figure-1:**
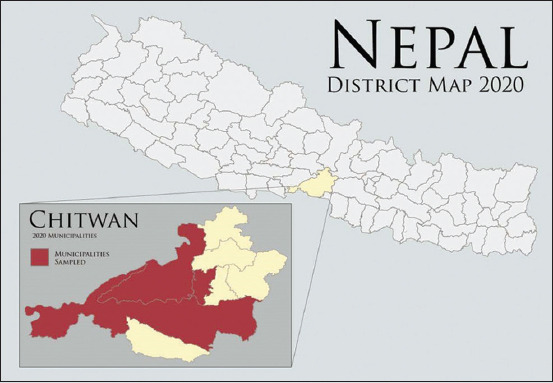
District map of Nepal (as per new political map of Nepal approved by the upper house of Nepal’s Parliament in 2020) highlighting the district of study and rural municipalities. Border within Chitwan is based on municipality borders from 2020.

This cross-sectional study was conducted in the Chitwan district, located in southwestern part of Narayani Zone, Central Development Region of Nepal with Latitude: 27° 34’ 59.99” N and Longitude: 84° 30’ 59.99” E (Figure-1). All samples were collected in May and early June, 2019. Chitwan district has emerged as a major income-generating enterprise in the agricultural sector over the past 35 years, contributing 39.5% of Nepal’s poultry population, 21.8% of poultry meat production, and 36.5% overall egg production [15,16].

### Design: Data collection and sampling

Based on the review of district documents on poultry production and meat marketing, Ministry of Livestock production documents, previous research and personal communications, purposive sampling of 18 commercial poultry farms (ten broilers and eight layers) was made. Farms were categorized based on size, type (broiler/layer), and geographical location – the samples were collected from 13 major villages and towns of the municipalities. Farms of different sizes were selected to ensure an adequate representation of the poultry industry. Farms were categorized into small (200-500 birds; n=4), medium (501-1000 birds; n=6), and large (>1000 birds; n=8) farms. Not every village in the district had all sizes and types of commercial poultry farms present. Within the farm size, type, and geographic location categories, random number selection was used to select farms for sampling. Selected farms were in various stages of production and in good health at the time of sampling. Samples were collected over an 18-day period, (May 17-June 3, 2019). A total of 288 environmental samples were collected from various sources, as shown in Table-1. The number of samples was dictated by budget restrictions. Sample number and type decisions were based on prior research studies and designed to provide a robust opportunity to identify the presence of NTS, if present on a farm. Within the limit of 288 samples, we restricted the number of farms to 18. Thus, 18 became the unit of analysis for the farm management survey portion of the study.

**Table-1:** Number of environmental samples collected by source and farm size.

Environmental samples	Four small farms (200 500 birds)	Six medium farms (501 1000 birds)	Eight large farms (>1000 birds)	Total
Water (n = 1, 2, or 3)^a^	4	12	24	40
Soil/Litter/Bedding (n = 2, 3, or 4)^a^	8	18	32	58
Adult bird feces (n = 2, 3, or 4)^a^	8	18	32	58
Young bird feces (n = 2, 3, or 4)^a^ (only in mixed age populations)^b^	4	0	4	8
Feed (n = 2, 3, or 4)^a^	6	18	32	56
Farm swabs (n = 1, 2, or 3)^c^	4	12	24	40
Eggshell swabs (n = 2, 4, or 6)^d^	2	8	18	28
Total Samples	36	86	166	288

a Environment samples represented by name and parentheses represents number of samples from each small, medium, and large farm, respectively.

b Additional feces were collected on farms with mixed age groups.

c Farm swabs were taken from three different poultry contact surfaces within the poultry house.

d Eggshell swabs were taken only from layer farms with birds in laying stage

A questionnaire including open- and closed-ended questions was prepared, pilot-tested with one non-participating farm manager then revised. All questionnaires were administered by the same two veterinarians to ensure consistency and translated into the local language during survey administration. The questionnaire included data concerning farm, poultry and poultry house characteristics, management of birds, biosecurity practices, presence of domestic animals and wildlife, feeding and watering practices, cleaning and disinfection procedures, vaccination and antibiotic use, and more. If farm biosecurity protocol prevented the veterinarians from entering the poultry houses, the farm staff was verbally instructed on how to collect samples and observed through the fence. “Farm” swabs involved wetting the swab in sterile water and streaking three different poultry-contact surfaces within the poultry house and egg swabs involved swabbing three different eggshells. The end of the swab was then broken off into 25 mL of sterile water. For all other samples, approximately 25 g of material (bedding, feces, feed, and water) in contact with poultry, were randomly collected throughout the farm.

### Testing: Isolation and identification of NTS enterica

Samples were pre-enriched with 1:9 concentration of buffered peptone water at 37°C for 18-20 h after preparation of the original homogenate in accordance with ISO 6579 protocol for pre-enrichment [17]. For each sample, 2 mL of the pre-enriched homogenate was then transferred to another sterile container and sent to the Laboratory of National Zoonoses and Food Hygiene Research Centre (NZFHRC) in Kathmandu, Nepal, for selective culture, isolation and biochemical analysis, and antimicrobial resistance testing on positive cultures.

Isolation of *Salmonella spp*. was carried out according to protocol ISO 6579 [17] with slight modifications, used for selective culture and biochemical identification. Pre-enriched samples were inoculated in Rappaport-Vassiliadis (RV) broth at 41°C for 24 h and *Salmonella shigella* agar was used for selective culture. Suspected colonies were subcultured and isolated colonies were cultured on nutrient agar slants for further identification and biochemical characterization.

The initial identification step was done using Gram’s stain and oxidase test; all isolates showing Gram’s stain positive and/or oxidase-positive were discarded. Then, other isolates were biochemically tested using Indole, Methyl red, Voges–Proskauer, Citrate utilization, Triple sugar iron (TSI), and urease tests as per the protocol described by Ewing [18]. The colonies showing *Salmonella* specific IMViC pattern (− + − +) were further inoculated on TSI slants, and colonies that produced alkaline slant (pink) and acidic butt (yellow) with or without H_2_S production (blackening) were tested for urea hydrolysis on urea agar slants. All the urease negative isolates were considered as biochemically confirmed *Salmonella* isolates. Positive isolates were submitted for serovar testing by Kauffmann-White-Le Minor scheme at the Nepal Agricultural Research Council in Lalitpur, Nepal.

### Statistical analysis

Microsoft Excel 2016^®^ (Microsoft Corporation, WA, USA) was used for data entry and management and IBM SPSS v25 (IBM Corp., NY, USA) was used for statistical analysis. The map of the sample area was created withArcGIS v10.3.1 (ESRI, CA, USA) using administrative borders fromHumanitarian Data Exchange v.1.43.5 (available at https://data.humdata.org/dataset/administrative-bounadries-of-nepal) and edited with Adobe Illustrator v16 (Adobe Inc, CA, USA). Farms were identified as positive based on at least one environmental sample collected on the farm testing positive for NTS. Due to the small sample size (n=18) variables were collapsed into two categories for association testing. To measure the impact of each factor individually on the presence of NTS on the farm, a two-tailed Fisher’s exact test was used. Odds ratio (OR) and 95% confidence intervals (CI) were calculated. Values of p<0.05 were considered statistically significant. Due to the small sample size, statistical significance was difficult to reach and some risk factors that were not statistically significant were included in the discussion as they may offer valuable information and be of interest in future studies.

## Results

### Farm-level NTS prevalence by farm type and size

Ten (55.5%) of the 18 poultry farms were found to have at least one environmental sample test positive for NTS. The point prevalence rate varied according to the type of farm: 80% (8 of 10) of broiler farms and 25% (2 of 8) of layer farms were positive; and by size of farm: 75% (6 of 8) large farms 0% (0 of 6) medium and 100% (4 of 4) small farms were found positive for NTS.

### Environmental sample testing

Out of the total 288 samples taken, 9% (26/288) were positive. The isolation rate varied according to the origin of samples: 27.5% (11 of 40) of water samples; 10.6% (7 of 66) feces samples; 8.6% (5 of 58) of bedding samples; 5% (2 of 40) of farm swabs (n=40); 1.8% (1 of 56) of feed samples; and 0% (0 of 28) of eggshell swabs were positive. Note: Serovar testing data are presented in the 2^nd^ paper in this series (www.veterinaryworld.org/Vol.14/February-2021/15.pdf).

### Farm questionnaire summary results

Farms were selected based on type (broiler and layer) and size and distributed geography. Detailed farm questionnaire results can be found in Table-2. Within these selection parameters, most of the farm owners were male (83%) and all were literate with 22% having completed graduate-level education. Most (89%) poultry houses had concrete floors and none were fully enclosed. Most (56%) of the farms were exclusively poultry (no other domestic animals) and half reported seeing wildlife near their poultry houses daily. All farmers reported using rice husk bedding, most (72%) reported using pelleted feed (vs. mash) and most (94%) reported feeding birds manually. None of the farmers were breeding their own birds nor were they marketing directly to consumers. Over half the farms (61%) were following all-in/all-out flock management while 39% were mixing ages.

**Table-2:** Summary of farm questionnaire results (n=18 farms).

Owner information	Number	%
Gender of farm owner		
Male	15	83%
Female	3	17%
Owner education level		
Illiterate	0	0%
Primary	7	39%
Secondary	7	39%
Graduate	4	22%
Age of farm owner		
35 years or younger	6	33%
36 50 years	6	33%
51 years or older	6	33%
Owner experience in poultry		
10 years or less	10	56%
11 years or more	8	44%
Gender of primary caretaker		
Male	6	33%
Female	4	22%
Both	8	44%

**Facility information**	**No.**	**%**

Distance of farm to the road		
100 m or less	7	39%
101 350 m	5	28%
351 m or more	6	33%
Distance to the nearest farm		
100 m or less	6	33%
101 200 m	7	39%
201 m or more	5	28%
Floor material		
Concrete	16	89%
Clay/Dirt	2	11%
Cage	0	0%
Style of chicken house		
Two Sides Open	7	39%
Three Sides Open	5	28%
Four Sides Open	6	33%
Closed	0	0%
Age of the chicken houses		
4 years or less	6	33%
5 11 years	6	33%
12 years or older	6	33%
Other domestic animals present		
No	10	56%
Yes	8	44%
Frequency wildlife observed near poultry		
Frequently (every day)	9	50%
Semi frequently (once per week)	3	17%
Rarely (once/mo)	4	22%
Never	2	11%

**Feed, water, bedding information**	**No.**	**%**

Type of bedding		
Rice Husks	18	100%
Type of feed		
Pellet	13	72%
Mash	5	28%
Style of feeding		
Automatic	1	6%
Semi automatic	0	0%
Manual	17	94%
Source of water for farm		
Tank	7	39%
Tap	9	50%
Well	2	11%
Surface	0	0%
Water delivery to poultry		
Nipple type	2	11%
Bell type	15	83%
Open	1	6%

**Poultry information - general**	**Number**	**%**

Poultry breeding on farm		
No	18	100%
Type of flock		
Layer	7	39%
Broiler	10	56%
Mixed	1	6%
Destination of meat/eggs		
Consumer direct	0	0%
Retailer	6	33%
Wholesaler	4	22%
Mixed	8	44%
Collector	0	0%
Feed restriction prior to sale		
No	17	94%
Yes	1	6%
Poultry cohort management		
All in all out	11	61%
Staggered/mixed ages	7	39%

**Poultry health information**	**No.**	**%**

Frequency of vet/paravet visit		
Weekly	5	28%
> once/year	5	28%
Once/year	7	39%
Never	1	6%
Medical records kept		
Yes	9	50%
No	9	50%
Vaccination frequency		
At hatching	2	11%
Regularly	10	56%
Both	6	33%
Vaccine administered by		
Farm owner	4	22%
Employee	1	6%
Vet or paravet	1	6%
Mixed	11	61%
N/A	1	6%

**Farm biosecurity**	**No.**	**%**

Type of PPE used		
Boots	16	89%
None	2	11%
Quarantine area		
No	10	56%
Yes	8	44%
Handwashing area		
Yes	18	100%
Soap present at handwash station		
Yes	16	89%
No	2	11%
Disinfectants present		
Yes	9	50%
No	9	50%
Footbath used		
No	16	89%
Yes	2	11%
Cleaning frequency		
Deep clean after slaughter	7	39%
Spot cleaning weekly	1	6%
Both	8	44%
No cleaning	1	6%
Time the house is kept empty between flocks		
10 days or less	6	33%
11 21 days	5	28%
22 90 days	7	39%
Litter disposal location		
Off site	6	33%
Onsite near housing	8	44%
Onsite away from housing	4	22%

Only one farm reported never having a veterinarian or a paravet visit the farm, and five farms (28%) reported weekly visits; half of the farmers were keeping medical records, and all birds were reported as being vaccinated at some point in time although the researchers could not determine exactly which vaccines were being used when. Common *S. enterica* specific vaccination practices in the Chitwan area for commercial layer farms include using live vaccine at 6 weeks of age and killed vaccine at 15 weeks of age. Broilers are not commonly vaccinated for S. enterica. S. enterica serovars Gallinarum, Enteritidis, and Typhimurium are included in commonly used vaccines in the Chitwan District.

Biosecurity practices include having an area available for handwashing (100%) with soap present on most (89%) and wearing boots (89%). Disinfectant was present on half the farms and two farms (11%) were using footbaths. Note: Antibiotic use and administration practices, as well as antibiogram data, are presented in the 2^nd^ paper in this series.

### Risk factors

Although this study is descriptive in nature, some analysis was possible. The results of selected Fisher’s Chi-square (two-tailed) tests show several possible associations between farm practice/risk variables and finding NTS on participating farms. These variables plus additional variables of interest are summarized in Table-3 (non-modifiable factors) and Table-4 (modifiable factors). Among the non-modifiable factors, “Farm Type” and “House Type” showed an association with finding NTS on the farm. Farm Type – boiler operations (vs. layer) were found to have a higher odds of being positive for NTS (OR 12, 95% CI 1.3-111.3) as were having poultry houses with only two sides open (vs. three or more sides open) (OR 10.5 95% CI 0.9-121.4). Among the modifiable risk factors, only using pelleted feed (vs. mash) showed a notable association (OR 9, 95% CI 0.8-108.3). No associations were found to be statistically significant at the p<0.05 level. This vast amplitude in the CI may be due to small sample size. Additional association testing results are provided for variables considered to be potential risk factors or protective factors for *S. enterica* presence on poultry farms in general, and these potential (but not statistically significant) findings may indicate the need for additional research to clarify the relationship between variables.

**Table-3:** Associations between non modifiable risk factors and finding NTS on farms.

Variable/Risk factor	NTS +	NTS	Odd ratio (95% CI)	p value
Farm type				
Broiler	8	2	12 (1.3 111.3)	0.05
Layer	2	6		
House type				
Two sides open	6	1	10.5 (0.9 121.4)	0.07
Three or more sides open	4	7		
Education Level of farmer				
Secondary or Higher	8	3	6.7 (0.8 55.0)	0.14
Primary only	2	5		
Flock size				
Large (1200 or more)	6	2	4.5 (0.6 34.6)	0.19
Small (<1200)	4	6		
Frequency of wildlife/rodent/Pest seen				
Rare to never	4	8	4 (0.5 33.3)	0.32
Weekly to daily	4	2		
Age of houses				
Over 10 years old	6	3	2.5 (0.4 16.9)	0.64
<10 years old	4	5		
Age of farm owner				
Under 45 years	6	3	2.5 (0.4 16.9)	0.64
45 years old or above	4	5		
Age of chickens				
Younger than 60 days	6	3	2.5 (0.4 16.9)	0.64
60 days or older	4	5		
Floor material				
Clay/Soil	1	0	2.7 (0.1 75.1)	1.00
Concrete	9	8		
Water delivery method				
Others	2	1	1.75 (0.1 23.7)	1.00
Bell type waterer	8	7		
Distance from farm to road				
<100 m	4	3	1.1 (0.2 7.5)	1.00
More than 100 m	6	5		
Source of Water				
Municipal/Tap	5	4	1.0 (0.2 6.4)	1.00
Well/Tank	5	4		

**Table-4:** Associations between modifiable risk factors and finding NTS on farms.

Variable/Risk factor	NTS +	NTS	Odd ratio (95% CI)	p value
Food Type				
Pellets	9	4	9 (0.8 108.3)	0.12
Mash	1	4		
Management style				
Mixed/Staggered age	5	2	3 (0.4 22.7)	0.37
All in/All out	5	6		
Presence of other domestic animals				
No	7	3	3.9 (0.5 27.8)	0.34
Yes	3	5		
Antibiotic use				
Yes	9	5	3.6 (0.3 50.3)	0.54
No	1	2		
Frequency of cleaning				
Frequent/spot cleaning	7	2	5.8 (0.7 48.9)	0.35
Infrequent/between flocks	3	5		
Houses empty between flocks				
>2 weeks	7	4	2.3 (0.3 16.2)	0.63
2 weeks or less	3	4		
Keeping medical records				
Yes	5	4	1.0 (0.2 6.4)	1.00
No	5	4		
Use of PPE (boots)				
No	2	1	1.8 (0.1 2.4)	1.00
Yes	8	7		
Use of footbath				
No	9	7	1.3 (0.1 25)	1.00
Yes	1	1		
Frequency of visit by Vet/Paravet				
More than 1/year	6	4	1.5 (0.2 9.8)	1.00
Annually or less frequently	4	4		

## Discussion

Although there are many studies focused on the prevalence of NTS in poultry meat, slaughterhouse samples and postmortem samples of birds in Nepal [19-23], there are very few focusing on the prevalence of NTS from environmental samples of poultry farms.

Of the 288 environmental samples taken, 26 (9%) were found to be positive for NTS. This is only slightly lower than a previous study done in the Chitwan district by Dhakal and Manandhar in 2005 [24], who showed 12% NTS positive samples from the litter, food, and water from poultry farms in the Chitwan district. Some factors that might account for this variation include region sampled, methods used for sample collection and testing, types of facilities chosen for sampling, and the season in which testing was performed. When compared with the research conducted in a neighboring country, India, which has similar poultry rearing practices to Nepal, the frequency of isolating *S. enterica* in the environmental samples ranged widely from 7.9 to 95.7% [25]. The sample collection period for this study was in the pre-monsoon season with generally dry and extremely hot weather with average maximum daytime temperature being 30.9°C.

The prevalence of NTS found in our study varied depending on the source of the environmental sample with a high of 27.5% (water) to a low of 0% from eggshell swabs. Similar research from Algeria showed a lower prevalence of *Salmonella* among environmental samples; 2.18% of water samples, 3.12% of feces samples, 3.93% of farm swabs, and 6.25% for wipes, while all the feed samples were free of *Salmonella* [26]. Research from Nigeria showed *S. enterica* prevalence to be; feces (23%), feed (22.7%), litter (20.3%), and dust (18.9%) with water samples having the lowest prevalence at 15.1% [27]. Another study from North Carolina, USA found the prevalence of *Salmonella* in fecal samples was 38.8%, feed was 27.5% while none of the water samples were positive [28]. Interestingly, our study found water to have the highest rate of positivity at 27%. In addition, for two of the study farms, water was the only positive environmental source on the farm. This was unexpected given the results from the above-mentioned studies measuring similar parameters and introduces the question of water being a potential source of introduction of NTS onto the farm. The unexpectedly high prevalence in water may also be due to the collection of water samples directly from the water feeder which may have collected biofilms or contamination from the environment. This discrepancy could also be the result of water from the feeders not being processed through any filter or chlorinated to remove environmental contaminants. In addition, the level of contamination in water seemed to be unaffected by other factors such as layer/broiler farm type, water source, cleaning regimen, or biosecurity protocols. Because water samples were not expected to test positive at this rate, survey questions surrounding water administration were limited to water source and feeder type, and did not include well type, cleaning protocols for water dispensers, and frequency the water is changed or the distance from the water feeders to the ground which could result in contamination. The fact that these samples were taken in the dry season could also have an impact, as the moisture around the poultry waterers could provide a more hospitable environment for NTS growth and biofilm accumulation when compared with the dry, hot surroundings.

In this study, the prevalence of NTS isolates from all eggshell swabs was negative. This was also an unexpected result as previous research has shown eggshell contamination to be commonplace [29-35]. There are several possible explanations for this discrepancy. Not all the layer farms tested were in the egg production stage, reducing the number of farms from which eggshell swabs could be taken. In addition, all but one of the layer facilities that were producing eggs, were negative on all other samples, suggesting a low prevalence of NTS among layer farms. The small sample size makes it difficult to draw any definitive conclusions.

## Non-modifiable risk factors

### Broilers versus layers

Samples taken from broiler farms were far more likely to be contaminated with *S. enterica* than samples taken from layer farms (OR=12; 95 CI 1.13-111.3 and p=0.05). These findings are similar to research conducted in other countries where broiler flocks were found to be positive for *S. enterica* at higher rates than layer flocks [36]. A possible explanation suggested by previous researchers could be a difference in procedures for cleaning and disinfection of broiler farms when compared with layers [36-38]. As broilers have a shorter period of rearing (around 36-50 days in Nepal), farmers are trying to maximize the number of rearing cycles per year and minimizing the time between cycles. This might lead to a shorter time available for cleaning and disinfection before new stock is introduced to the farm, making it more difficult to follow the “all in-all out” management principle [36,39]. In this study, we found 64% of all farms reported practicing all-in/all-out management. Additional factors contributing to NTS contamination could be that the material used for construction of the house may not allow for satisfactory cleaning and house design may allow bacterial contamination from surrounding wildlife such as insects or rodents. The ability of *S. enterica* to resist desiccation allows it to survive for long periods in the environment [40,41].

The lower prevalence of infection found in layer flocks could be due to the declining rate of *S. enterica* colonization and fecal shedding 2 weeks post-infection in laying chickens from pullet and layer flocks [35]. Moreover, in some farms, the birds may be infected with *S. enterica* without showing signs of the illness, which means the presence of a sub-clinical infection in the flock. Feces from these flocks may contain *S. enterica* in low numbers [41]. In addition, vaccination against *S. enterica* in layer flocks (but not in broiler flocks) in Nepal may also lead to lower rates of infection. These vaccines are less common among broiler farms, although our current survey did not reveal any specific link between our nonspecific “vaccination” variable and NTS prevalence.

#### Poultry house design

Samples taken from poultry houses with two sides open were more likely to be contaminated than houses with three or more sides open which might be attributed a lack of adequate ventilation (OR=10.5; 95 CI 0.9-121.4 and p=0.07). Various studies suggest that infection could occur by oral ingestion of dust from external surfaces that can be contaminated by airborne movement during the time of feeding or pecking [42,43]. There are also other findings showing a relationship between low rate of airflow causing dead pockets to be formed in litter/manure and increasing the counts of *S. enterica* [44]. It has been shown that *S. enterica* remains in the dust of ventilation filters for several months [45]. Other studies have suggested airflow directly impacts litter dampness and that broiler growth improves with improvement of ventilation [44,46-48].

#### Education level of farmer

Another non-modifiable risk factor considered was the education level of the farmer (OR 6.67; 95 CI 0.8-55.0 and p=0.07). It is interesting to note that the farmers with higher education were more likely to have NTS contamination on their farms. One possible explanation could be that farmers with higher levels of education may be less inclined to spend their time on the farm and farm management may be relegated to other people with less stake in the health of the flock. People with higher education may also have other sources of income, splitting their responsibilities between farming and other off-farm duties. The results could also be due to spurious causes. For example, higher educated farmers may be more likely to own broiler farms, and the contamination is due to broiler management and unrelated to the level of education directly. Additional research related to education of farm owners, managers, and workers would be needed to properly explore this finding.

### Modifiable risk factors

#### Pelleted feed

Due to small sample size (n=18 farms), risk factors could not be strongly correlated with any specific modifiable practice. However, there were two factors of interest that may warrant further study. Among poultry farms feeding pellet food, 69% of these farms (9 of 13) had at least one environmental sample positive for *S. enterica* compared to 20% of farms feeding mash (OR 9; 95 CI 0.8-108.3 and p=0.12). It is important to note that these test results do not correlate to farms with feed that tested positive, but any environmental sample. Only 1 of the 56 feed samples tested positive and this feed was mash. It is also important to note that the sample size is very small, particularly in the number of farms feeding mash. It is possible, for example, that pellet food is a more popular feed for broiler farms, and other factors related to broiler poultry management are responsible.

The incidence of *S. enterica* in poultry feed and feed ingredients is known to be highly variable ranging from 0 to 78% [49-51]. Various factors including source and quality of feed ingredients, and storage conditions could contribute to this variation. The recovery of *S. enterica* in feed using meat and bone meal has been described in the previous studies [49]. Higher incidence of *S. enterica* in meat and bone meal could be due to improper sterilization of these ingredients and suggest that they may be the major source of *S. enterica* in compound feed. In Nepal, mash feed prepared by the farmer at the farm level usually do not include bone or meat meal, but pellet feed prepared in industry level uses it regularly. Further feed analysis could reveal meaningful data, but larger samples sizes would be needed as well as specific feed composition and origin. The previous studies also showed that *S. enterica* has been recovered from dry products even after they have been processed at relatively high temperatures [52]. This could be the result of post-processing contamination or the ability of *S. enterica* to survive extremely well when heated in media of reduced water activity [52-54]. Dry animal feeds can also play an important role in the epidemiology of human salmonellosis [55]. Another study on *S. enterica* contamination in US swine feed reported higher pathogen presence in pelleted commercial feed when compared to on-farm mixed mash products [56].

#### Poultry cohort management

The other factor worth noting was the farm management style. Samples taken from farms who had mixed age chickens on site had higher odds of having positive isolates of *S. enterica* than farms that practiced all-in all-out style of management, where all the chickens managed on the farm enter and leave at the same time (OR=3; 95 CI 0.4-22.7 and p=0.37). This finding agreed with the previous studies looking at the effect of multiage management on the farm with the occurrence of *S. enterica* [57-59].

All other modifiable risk factors including biosecurity measures, cleaning procedures, frequency of veterinary visits, and presence of other domestic animals on the farm did not show a significant relationship to finding NTS on the farm. This is likely due to several factors. Many of the poultry farms visited had similar biosecurity measures. Most had separate boots for entering the chicken house. Most farms used similar protocols for cleaning, which included a mixture of VirkonS, lime powder, fumigation, and incineration. This lack of variation provided no negative grouping for comparison.

As previously mentioned, the major limitation of this study is the small number of farms available for analysis. Collecting samples during the dry season also may have decreased the likelihood of finding positive environmental samples. However, the inclusion of multiple farm sizes and types from 13 villages provides a good snapshot of the current farm practices in this important and rapidly expanding industrial hub.

## Conclusion and Recommendations

To the best of the authors’ knowledge, this is the first systematic, descriptive study with a comprehensive survey to evaluate the various risk factors that may contribute to NTS acquisition, maintenance, and spread among Nepal’s poultry farms. This study provides important baseline data on the prevalence of NTS among 18 poultry farms located in 13 villages in Chitwan, Nepal’s leading poultry producing district. The in-depth questionnaire data provide a picture of common poultry farming practices, biosecurity measures, and farmer demographics for this region. Although the small sample size precluded robust analysis, some interesting trends emerged that warrant further investigation. This descriptive cross-sectional study suggests some farm management practices that may reduce the likelihood of acquiring and maintaining NTS on the farm. This data will be useful to improve farm management practices, direct future epidemiological studies, and ultimately reduce the spread of zoonotic strains of NTS from poultry to humans.

For ensuring *S. enterica* -free feed and water, regular microbiological examination of feed and water should be done to estimate the microbial load so that use of sanitizers/ additives can be done promptly. Furthermore, regular, and proper cleaning of feed and water containers is important to reduce biofilm formation. Proper ventilation also ensures less growth of microbes, which can reduce the chances of infection.

NTS contamination was higher among broiler operations than layer operations. Broiler farmers should take more care during cleaning and disinfection. The risk of NTS contamination was also higher when flocks were reared on farms with multiage management rather than on farms with all-in/all-out management practice. Applying “all in-all out” procedures, with appropriate biosecurity measures against animated (owner, worker rodent/pest/wild animals) or unanimated vectors (dust, litter, feed, and water) can be effective to reduce contamination of *S. enterica* on the farm. However, further detailed studies on the correlation of levels of biosecurity and incidence of *S. enterica* in poultry farms in this region of Nepal are needed.

Finally, there are some other important risk factors which were not considered in this research but should be considered in future studies. Risk factors to explore include: Biofilm development in farm equipment, vertical transmission of *S. enterica*, feed composition, rodent/pest control strategies, the role of temperature and humidity, cleaning and disinfecting practices with relation to the previous infection in the same poultry house, and seasonality at the time of sample collection. Most of these risk factors have been already studied in other countries, but not yet in Nepal.

Serotyping results and antibiotic resistance profiles of *S. enterica* isolates can be important to fully understanding the epidemiology, ecology, and distribution of this pathogen in the poultry farms of Nepal (see accompanying paper). Repeating this study would help estimate the patterns and distributions over time, including data about emerging serotypes and provide information on epidemiology that can help design future mitigation plans.

It can be concluded that there is a high prevalence of NTS found in environmental samples collected from the poultry farm environment in the Chitwan district of Nepal. Therefore, infection of birds with NTS from environmental sources is highly possible if no additional preventive strategies are taken. The present study was designed to be descriptive in nature and revealed fairly consistent farm practices within the Chitwan region. Regarding farm management practices, there is room for improvement regarding the use of footbaths, flock cohort management, cleaning and disinfection protocols and medical record keeping, making these easy targets for farmer/worker education campaigns.

Finally, findings from this study underlines the need for a “One-Health” approach that uses human, animal, and environmental health resources to solve this dynamic issue at the intersection of the human-animal-environment interface to avoid more human illness and diseases along with future outbreaks.

## Authors’ Contributions

SS1 and PDF actively worked on the sample collection, questionnaire design and initial sample processing and draft writing. DKP supervised sample handling and completed the process of identification and isolation of *S. enterica* strains. PF and MW participated actively in study design and data analysis, while SS2 contributed in part to the study design, draft writing, data interpretation, and revising along with SS1, PF, and MW. SS2 and MJW carefully monitored each level of research. All authors read and approved the final manuscript.
